# Renin-angiotensin system inhibitor use and cardio-renal outcomes in non-proteinuric chronic kidney disease: a post-hoc analysis of the Frontier of Renal Outcome Modification-Japan study

**DOI:** 10.1038/s41440-025-02536-x

**Published:** 2026-01-14

**Authors:** Hirohito Sugawara, Kiryu Yoshida, Chie Saito, Yoshinori Saito, Masanori Kato, Akihiko Kato, Ichiei Narita, Shoichi Maruyama, Jun Wada, Takashi Wada, Masahiro Yamamoto, Hidetoshi Ito, Kunihiro Yamagata, Hiroaki Ogata

**Affiliations:** 1 Division of Nephrology, Department of Internal Medicine, Showa Medical University, Northern Yokohama Hospital, Yokohama, Japan; 2 Department of Nephrology, Faculty of Medicine, University of Tsukuba, Tsukuba, Japan; 3 Department of Nephrology, Kosai Municipal Hospital, Kosai, Shizuoka, Japan; 4 Niigata Institute for Health and Sports Medicine, Niigata, Japan; 5 Department of Nephrology, Nagoya University Graduate School of Medicine, Nagoya, Japan; 6 Department of Nephrology, Rheumatology, Endocrinology and Metabolism, Okayama University Graduate School of Medicine, Dentistry and Pharmaceutical Sciences, Okayama, Japan; 7 Department of Nephrology and Rheumatology, Kanazawa University, Kanazawa, Japan; 8 Department of Medical Education, Showa Medical University School of Medicine, Tokyo, Japan

**Keywords:** Cardiovascular events, Chronic kidney disease, Non-proteinuria, Renin–angiotensin system, Implemental hypertension

## Abstract

Patients with chronic kidney disease (CKD) frequently experience cardiovascular events, and as per current therapeutic guidelines, renin-angiotensin system inhibitors (RASi) can protect the cardiovascular system in those with proteinuric CKD. Effectiveness of RASi in treating non-proteinuric CKD is still unknown, yet. In order to evaluate the impact of RASi on cardiovascular morbidity and mortality in patients with non-proteinuric CKD, we performed a post-hoc analysis of the Frontier of Renal Outcome Modification-Japan study. A urine protein-to-creatinine ratio less than 0.15 g/g or negative/trace protein on urinalysis was considered as non-proteinuric CKD. Those who have undergone dialysis, kidney transplant recipients, and patients who refused to give their consent were excluded. A composite of cardiovascular events, initiation of renal replacement therapy, and all-cause mortality was studied as the primary outcome. Of 2379 patients with CKD, 630 met the criteria for non-proteinuric CKD. Among them, 490 used RASi, and 140 did not. Although the RASi group was considerably younger and had a higher prevalence of hypertension and calcium channel blocker use, baseline characteristics were comparable. 12.1% of the control group and 16.7% of the RASi group experienced the primary outcome during follow-up, with no significant difference (adjusted HR: 1.37; 95% CI: 0.81–2.31). Secondary outcomes and analyses of RASi use for the whole observation period did not show any significant differences (adjusted HR: 0.81; 95% CI: 0.43–1.56). These results imply that RASi was not linked to a decreased risk of mortality or long-term events in those with nonproteinuric CKD.

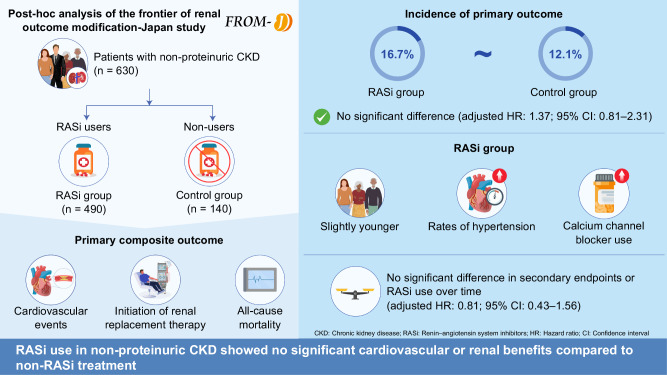

## Introduction

Chronic kidney disease (CKD) is a global health concern and a leading cause of cardiovascular disease (CVD) morbidity and mortality [1, 2]. Early intervention is essential. Proteinuria is a well-known risk factor for adverse cardiorenal outcomes, alongside age, underlying renal disease and CKD stage [3, 4]. A proteinuria threshold of 0.5 g/24 h is highlighted in the most recent Kidney Disease: Improving Global Outcomes (KDIGO) guidelines as clinically significant for determining the severity of CKD in both general and high-risk populations[5]. Patients with proteinuria have been the focus of the majority of clinical trials assessing treatments like renin-angiotensin system inhibitors (RASi), which include angiotensin-converting enzyme [ACE] inhibitors and angiotensin receptor blockers [ARB] [6, 7]. However, the prognosis and risk profile of patients with non-proteinuric CKD (i.e., proteinuria <0.5 g/24 h), who commonly visit renal clinics, remain poorly defined. This limitation is an important gap, as non-proteinuria is prevalent in the general population and across various clinical settings [8, 9].

The effects of metoprolol, amlodipine, and ramipril on the development of hypertensive kidney disease were assessed by the African American Study of Kidney Disease and Hypertension. The mean GFR slope difference between ramipril and amlodipine was less in those with UP/Cr ≤0.22, at 0.80 (0.43) mL/min/1.73 m²/year (*P* = 0.07). Importantly, there was no statistically significant interaction between the treatment effect and baseline proteinuria (*P* = 0.21). Baseline proteinuria did not significantly alter the decrease in clinical outcomes seen with ramipril (*P* = 0.25) [10].

The Frontier of Renal Outcome Modifications in Japan (FROM-J) study, a large prospective cohort investigating lifestyle interventions in CKD, offers a valuable opportunity to examine this understudied group [11]. Its broad inclusion of non-proteinuric patients and its representativeness of the Japanese CKD population make it well-suited to address this gap.

In this post-hoc analysis of the FROM-J study, we investigated the effects of RASi on cardiovascular morbidity and mortality in individuals with non-proteinuric CKD.

Point of view

- **Clinical relevance**: Our findings suggest that the cardio-renal benefits of renin–angiotensin system inhibitors (RASi) observed in proteinuric CKD cannot be simply extrapolated to patients with non-proteinuric CKD. This highlights the need for more individualized risk assessment and careful consideration of RASi prescription in this subgroup.
- **Future direction**: Prospective studies and randomized controlled trials focusing specifically on non-proteinuric CKD are warranted to clarify which patient profiles, if any, derive benefit from RASi therapy. Further work should also explore alternative therapeutic strategies and refine risk stratification tools for this growing population.
- **Consideration for the Asian population**: Because this analysis was conducted in a Japanese cohort, our results raise the possibility that the limited benefit of RASi in non-proteinuric CKD may also apply to Asian populations.

## Methods

### Study design and patients

The FROM-J study was a prospective cluster-randomized trial that included patients with CKD under the care of general practitioners in Japan, and was subjected to a post-hoc analysis. Details of the FROM-J study’s methodology and results have already been published [10].

For this analysis, we included patients with non-proteinuric CKD, defined as a urine protein-to-creatinine ratio <0.15 g/gCr. Urine protein measurements were obtained prior to the initiation of RASi therapy. Patients with proteinuria (>0.15 g/gCr), undergoing dialysis, post–renal transplantation, or those who did not provide consent were excluded.

Patients were stratified into two groups: the RASi group, which included patients who received RASi, and the control group, which included patients who did not receive RASi. The 0.15 g/gCr threshold was based on the KDIGO classification for identifying proteinuric CKD [5]. The classification of the two groups was based on the proteinuria level registered at the first visit. This visit represented the baseline visit of the present study.

Ethics Committee for Clinical Research at Showa Medical University approved the study(approval nos. J250425-012, J250425-013) and was carried out in compliance with the principles of the Declaration of Helsinki. For this secondary analysis, the ethics committee approved an exemption from obtaining individual consent.

### Outcomes

A composite outcome consisting of the following: CVD events, initiation of renal replacement therapy (RRT), and death from any cause was the primary outcome. Secondary outcomes were assessed for each outcome. Heart failure, non-fatal myocardial infarction, non-fatal stroke, and cardiovascular death were all considered CVD occurrences. Clinical outcomes were ascertained from clinical records and follow-up case report forms, and all events were reported by treating physicians. Non-fatal myocardial infarction was defined by ischemic symptoms with supporting electrocardiographic changes and troponin level. Stroke is characterized by a sudden, focal neurological dysfunction of vascular origin that lasts for at least 24 h. Heart failure was defined as hospitalization requiring intravenous diuretics. RRT was defined as kidney transplantation and chronic maintenance dialysis (hemodialysis, peritoneal dialysis), and the initiation of these treatments was evaluated.

### Statistical analyses

Continuous variables are reported as either means ± SDs or medians and interquartile ranges according to their distribution. Comparisons of variables between the two groups were performed using the unpaired Student’s *t* test or Mann–Whitney test. Categorical variables were analyzed using the chi-squared test.

In survival analyses, endpoints of interest were all-cause death, CVD, and ESRD (End Stage Renal Disease), defined as the start of chronic dialysis therapy or kidney transplantation. ESRD was diagnosed on the day of the first dialysis session or transplantation. Death certificates or hospital records were used to determine the date and cause of death. Follow-up ended on December 31, 2014. We used the inverse Kaplan–Meier approach for time-to-event analysis.

Hazard ratios and 95% CIs for outcomes were estimated using multivariate Cox proportional hazards models. Adjustments were made for baseline covariates identified a priori as risk factors, based on previous studies involving a similar CKD population. The models were constructed as follows: model 1: unadjusted; model 2: adjusted for age, sex, body mass index (BMI), diabetes mellitus (DM), estimated Glomerular Filtration Rate (eGFR); model 3: adjusted for all variables in model 2 + albumin, hemoglobin, potassium, use of statins, and mineralocorticoid receptor antagonists.

We performed a subgroup analysis adjusted for the following baseline covariates: age, sex, BMI, systolic and diastolic blood pressure, DM, eGFR, albumin, and potassium. A thorough case analysis was carried out with reference to missing variables.

*P *< 0.05 was considered statistically significant. Data were analyzed using R software version 4.4.2 (R Foundation for Statistical Computing, Vienna, Austria).

## Results

### Participants and follow-up

**Fig. 1:**
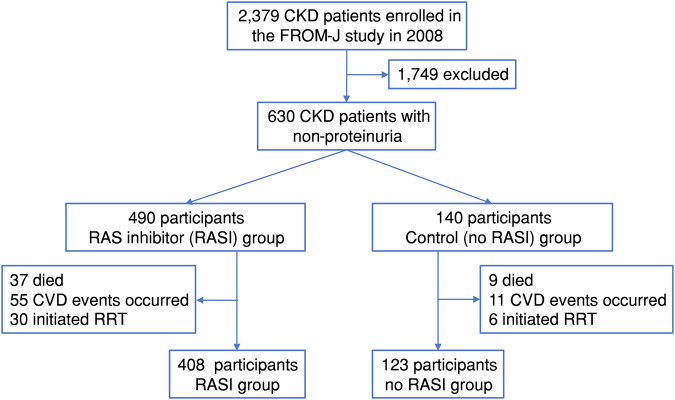
Flow diagram of participants. CKD chronic kidney disease, RASI renin-angiotensin system inhibitor, RRT renal replacement therapy

Between April 1, 2008, and October 19, 2008, 2379 patients with CKD, aged 40–74 years, were enrolled from 49 local medical associations in 15 different prefectures and followed up. Additional data collection and analyses, including patient-reported outcome measures, were performed to investigate the long-term outcomes of the intervention in the 10th year after the study. A total of 630 of those included had nonproteinuric CKD, and they were grouped into two groups: RASi (*n* = 490, 77.8%) and control (*n* = 140, 22.2%). (Fig. 1 and Supplementary Table 1).

Table 1 presents the baseline patient characteristics. Patients in the RASi group were younger (66.6 vs. 68.8 years). Additionally, male sex (69.2% vs. 70.7%), diabetes (50.6% vs. 47.8%), and the level of eGFR (44.7 vs. 46.0 ml/min/1.73 m^2^) were comparable between the groups. In the RASi group, a history of hypertension (96.3% vs. 73.9%, *P* < 0.01) and blood pressure (systolic: 134 ± 13 vs. 130 ± 12 mmHg, *P* < 0.01; diastolic: 77 ± 10 vs. 75 ± 9 mmHg, *P* < 0.01), as well as those taking calcium channel blockers (69.6% vs. 44.3%, *P* < 0.01), were higher. The level of uric acid (6.6 vs. 6.2 mg/dL) was higher in the RASi group.

**Table 1:** Clinical characteristics of the included participants

Characteristics	Overall	RASI	no RASI
*n*	630	490	140
Age, years	67.1 [41.1, 75.1]	66.6 [41.1, 75.1]*	68.8 [43.0, 75.1]
Male sex, no. (%)	438 (69.5)	339 (69.2)	99 (70.7)
Body mass index, kg/m^2^	25.5 ± 3.8	25.4 ± 3.8	25.9 ± 3.6
Smoking, no. (%)	107 (17.3)	82 (17.0)	25 (18.1)
Diabetes mellitus, no. (%)	313 (50.0)	247 (50.6)	66 (47.8)
Hypertension, no. (%)	572 (91.4)	470 (96.3)*	102 (73.9)
Dyslipidemia, no. (%)	432 (69.0)	342 (70.1)	90 (65.2)
Blood pressure, mmHg			
Systolic	133 ± 13	134 ± 13*	130 ± 12
Diastolic	77 ± 10	77 ± 10*	75 ± 9
Estimated GFR			
mean, mL/min/1.73m^2^	45.0 ± 11.1	44.7 ± 11.1	46.0 ± 11.1
Distribution, no. (%)			
45 to <60, mL/min/1.73m^2^	366 (58.1)	278 (56.7)	88 (62.9)
30 to <45, mL/min/1.73m^2^	186 (29.5)	150 (30.6)	36 (25.7)
15 to <30, mL/min/1.73m^2^	70 (11.1)	56 (11.4)	14 (10.0)
<15, mL/min/1.73m^2^	8 (1.3)	6 (1.2)	2 (1.4)
Serum potassium, mEq/L	4.5 ± 0.6	4.5 ± 0.6	4.5 ± 0.6
Hemoglobin, g/dL	13.5 ± 1.8	13.5 ± 1.8	13.5 ± 1.9
HbA1c, %	6.0 ± 1.0	6.0 ± 1.0	6.1 ± 1.2
MRA, no. (%)	18 (2.9)	14 (2.9)	4 (2.9)
Alpha blocker, no. (%)	40 (6.3)	34 (6.9)	6 (4.3)
Beta blocker, no. (%)	84 (13.3)	69 (14.1)	15 (10.7)
CCB, no. (%)	403 (64.0)	341 (69.6)*	62 (44.3)
Diuretics, no. (%)	55 (8.7)	38 (7.8)	17 (12.1)
Thiazide, no. (%)	58 (9.2)	49 (10.0)	9 (6.4)
Statin, no. (%)	239 (37.9)	189 (38.6)	50 (35.7)

*means *p* < 0.05

### Outcomes

**Fig. 2:**
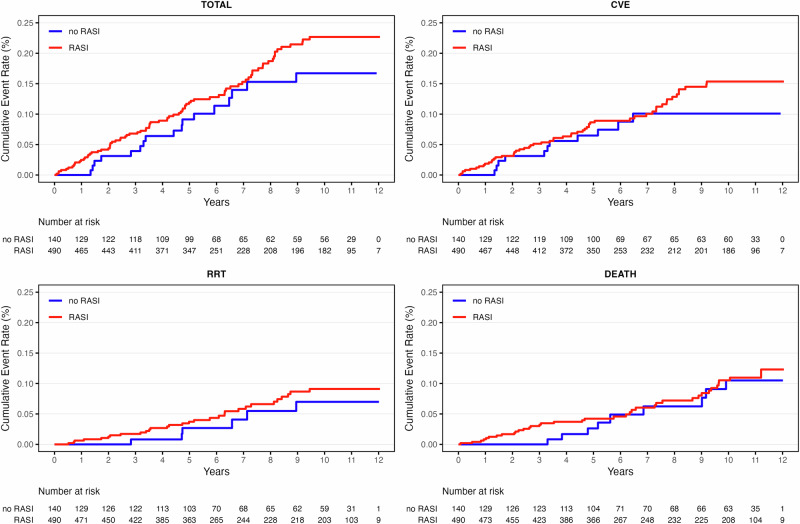
Primary and secondary outcomes. The primary outcome (**A**) was a cardiovascular disease (CVD) events (**B**), initiation of renal replacement therapy (RRT) (**C**), composite of a death from any cause (**D**)

In the RASi and no RASi groups, the primary composite outcome of all-cause death and CVD, ESRD, occurred in 82 participants (16.7%) and 17 participants (12.1%), respectively (HR, 1.37; 95% CI, 0.81–2.31; *p* = 0.235) (Table 2 and Fig.2A).

**Table 2:** Primary and secondary outcomes

Outcome	Model	RASI		no RASI		Hazard Ratio (95% CI)	*p*
		Total No.	Event No. (%)	Total No.	Event No. (%)		
Primary outcome							
	Model 1	490	82 (16.7)	140	17 (12.1)	1.37 (0.81–2.31)	0.235
	Model 2	476	81 (17.0)	133	17 (12.8)	1.41 (0.83–2.39)	0.201
	Model 3	328	52 (15.9)	99	12 (12.1)	1.32 (0.69–2.52)	0.402
Secondary outcomes							
All cause death							
	Model 1	490	37 (7.6)	140	9 (6.4)	1.17 (0.56–2.42)	0.674
	Model 2	476	36 (7.6)	133	9 (6.8)	1.25 (0.60–2.61)	0.552
	Model 3	328	25 (7.6)	99	7 (7.1)	1.06 (0.45–2.51)	0.888
Cardiovascular events							
	Model 1	490	55 (11.2)	140	11 (7.9)	1.43 (0.75–2.73)	0.279
	Model 2	476	54 (11.3)	133	11 (8.3)	1.55 (0.81–2.97)	0.190
	Model 3	328	31 (9.5)	99	8 (8.1)	1.34 (0.60–2.98)	0.476
Renal replacement therapy							
	Model 1	490	30 (6.1)	140	6 (4.3)	1.41 (0.59–3.38)	0.446
	Model 2	476	30 (6.3)	133	6 (4.5)	1.48 (0.60–3.61)	0.394
	Model 3	328	23 (7.0)	99	4 (4.0)	1.45 (0.49–4.28)	0.501

The secondary outcomes did not significantly differ between the groups. Altogether, 37 (7.6%) and 9 (6.4%) cases of any cause mortality occurred in the RASi and no RASi groups, respectively (HR, 1.17; 95% CI, 0.56–2.42; *p* = 0.674; Table 2 and Fig. 2B). Furthermore, in RASi group had incidence of CVE in 55 (11.2%) patients and 11 (7.9%) in the no RASi group (HR, 1.43; 95% CI, 0.75–2.73; *p* = 0.279; Table 2 and Fig. 2C). RRT events were reported in 30 (6.1%) patients from the RASi group and 6 (4.3%) from the no RASi group (HR, 1.41; 95% CI, 0.59–3.38; *p* = 0.446; Table 2 and Fig. 2D). The details of CVE are as follows: stroke (HR, 1.42; 95% CI, 0.54–3.71; *p* = 0.475), heart failure (HR, 1.85; 95% CI, 0.55–6.24; *p* = 0.323), and myocardial infarction (HR, 0.85; 95% CI, 0.27–2.62; *p* = 0.773) (Supplementary Table 2). Moreover, the results were similar, even after adjusting for confounding factors. Finally, we examined the effects of ACE inhibitors and ARB separately; however, no significant differences were observed (Supplementary Table 3A–B). On propensity score matching was performed, and details are as follows: HR 2.22 (1.01–4.88) *p* = 0.04(stratified by subclass) and HR 2.22 (1.11–4.42) *p* = 0.02 on covariate-adjusted (Model3) +cluster(subclass).

### Subgroup and sensitivity analyses

**Fig. 3:**
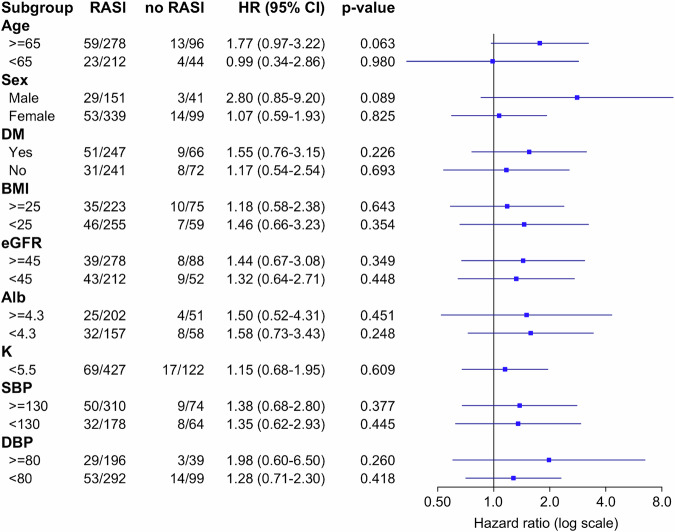
Forrest plot of subgroup analysis

Subgroup analysis showed no significant differences in primary and secondary outcomes (Fig. 3 and Supplementary Table 4A–C). Furthermore, because we considered that the prescription period might affect the effectiveness of RASi, we conducted a sensitivity analysis between the prescription and non-prescription groups over the entire duration. As in the previous analysis, no significant differences were observed (HR 0.81, 95% CI 0.43–1.56; *p* = 0.533; Table 3). Given prior reports suggesting that RASi suppress eGFR decline and reduce new-onset proteinuria, we also evaluated these endpoints but found no significant effects (Supplementary Table 5A–B). Moreover, the RASi group had a higher proportion of participants with high blood pressure and calcium channel blocker use. Therefore, we performed a multivariate analysis that included each factor; however, no significant changes were observed (Supplementary Table 6). In this study, we investigated whether there was a synergistic effect with RASi owing to early intervention; however, no significant results were found (Supplementary Table 7).

**Table 3:** Primary and secondary outcomes in prescription in the entire duration group

Outcome	Model	RASI		no RASI		Hazard Ratio (95% CI)	*p*
		Total No.	Event No. (%)	Total No.	Event No. (%)		
Primary outcome							
	Model 1	335	55 (16.4)	66	11 (16.7)	0.81 (0.43–1.56)	0.53
	Model 2	325	55 (16.9)	62	11 (17.7)	0.94 (0.49–1.81)	0.85
	Model 3	225	34 (15.1)	40	6 (15.0)	1.04 (0.42–2.57)	0.93
Secondary outcomes							
All cause death							
	Model 1	335	28 (8.4)	66	6 (9.1)	0.74 (0.31–1.79)	0.5
	Model 2	325	27 (8.3)	62	6 (9.7)	0.88 (0.36–2.17)	0.78
	Model 3	225	20 (8.9)	40	4 (10.0)	0.78 (0.26–2.37)	0.67
Cardiovascular events							
	Model 1	335	44 (13.1)	66	6 (9.1)	1.21 (0.51–2.84)	0.66
	Model 2	325	44 (13.5)	62	6 (9.7)	1.53 (0.65–3.64)	0.33
	Model 3	225	25 (11.1)	40	3 (7.5)	1.87 (0.52–6.72)	0.34
Renal replacement therapy							
	Model 1	335	13 (3.9)	66	5 (7.6)	0.41 (0.14–1.14)	0.09
	Model 2	325	13 (4.0)	62	5 (8.1)	0.76 (0.25–2.34)	0.64
	Model 3	225	10 (4.4)	40	3 (7.5)	1.00 (0.21–4.78)	1

We incorporated longitudinal blood pressure during follow-up using extended Cox models with time-varying covariates (monthly control <130/80 and <140/90) and time-in-range (TIR) metrics. Adjustment for BP control did not materially change the association between RASi and the composite kidney outcome (Supplementary Table 4A–C). In contrast, better BP control (higher TIR and on-treatment periods with BP < 130/80 or <140/90) tended to be associated with a lower hazard, although precision was limited by events. Furthermore, 52% and 69% of patients achieved BP < 130/80 within 180 and 365 days, respectively.

## Discussion

The contribution of non-proteinuric nephropathies to the global burden of end-stage kidney disease (ESKD) remains poorly understood. The molecular processes and risk factors that accelerate the progression of ESKD in this population have not been extensively investigated. While the efficacy of RASi is well established in proteinuric conditions such as diabetic nephropathy and glomerulonephritis [6, 7], its role in non-proteinuric CKD is uncertain due to a lack of clinical evidence. In this study, we examined the association between the development of CVD in patients with non-proteinuric CKD and the pathophysiology and long-term protective effects of therapy. Furthermore, we observed that among those with non-proteinuric CKD, baseline RASi treatment was not linked to a decreased risk of long-term events or mortality.

Proteinuria is a primary renal disease that may evolve into ESKD [3, 4]. However, proteinuria is often inconspicuous or absent as the most frequent cause of ESKD (renal sclerosis, polycystic kidney disease, pyelonephritis/tubule-interstitial disease, etc.) [5, 11]. Effectively delaying the progression of renal failure in individuals with CKD and proteinuria requires maintaining a high level of attention. To establish treatment plans that could benefit the great majority of CKD patients who eventually develop ESKD through molecular mechanisms other than proteinuria, however, more research is still required.

Irrespective of the patient’s diabetes status, sodium-glucose cotransporter (SGLT) 2 inhibitors lower the risk of heart failure events in all eGFR and albuminuria groups as well as the risk of CVD or heart failure hospitalization [12, 13]. Furthermore, the J-CKD-DB study showed that, in comparison to other hypoglycemic medications, SGLT2 inhibitors significantly decreased the yearly eGFR decline and renal composite outcomes in patients with non-proteinuric type 2 diabetes [14].

Historically, RASi have been effective in proteinuric CKD due to their effects on glomerular hemodynamics and structure. They reduce intraglomerular pressure by inhibiting angiotensin II, which normally causes efferent arteriole constriction [15]. This mechanism lowers protein leakage and helps preserve glomerular integrity by protecting podocytes and the basement membrane [16, 17]. RASi also exerts anti-inflammatory and anti-fibrotic effects, which contribute to renal protection [18, 19].

Our findings were supported by a meta-analysis of 42 RCTs, which showed that RASi decreased renal failure in proteinuric patients compared to placebo, but had no discernible effects in non-proteinuric patients. Furthermore, the analysis found no significant reduction in cardiovascular events in either group compared to other blood pressure-lowering agents [20]. Shulman et al. reported that, among patients with nonproteinuric CKD, baseline use of RASIs compared with other antihypertensive agents showed no difference in CVD or mortality risk. However, secondary analyses considering continuous adherence and cumulative exposure revealed a significant reduction in both CVD and mortality risk with RASIs [21].

The reason for the ineffectiveness of RASi in non-proteinuric CKD in this study is primarily attributable to the small effect of intraglomerular pressure. Proteinuria is often caused by increased intraglomerular pressure and changes in glomerular permeability [3, 22]; RASi suppresses proteinuria by reducing this pressure and improving permeability. However, increased intraglomerular pressure and abnormal permeability are not the main causes of non-proteinuric CKD, limiting the effects of RASi. In addition, in non-proteinuric CKD—such as interstitial nephritis and chronic pyelonephritis—renal function decline is primarily caused by damage to the renal tubules and interstitium, rather than damage to the glomerulus; therefore, pressure regulation and basement membrane protection effects at the glomerular level, which RASi act on, are not very effective. Consequently, the hemodynamic and structural targets of RASi may be limited. Additionally, the pathogenesis of non-proteinuric CKD may be driven by immune, inflammatory, and fibrotic pathways not adequately addressed by RASi [23, 24].

The FROM-J study was designed to evaluate strategies for slowing CKD progression through early intervention and coordinated care between general practitioners and nephrologists. The title of the research is “A study to examine the usefulness of a medical system to promote cooperation between family doctors/non-nephrologists and nephrologists to prevent the progression of CKD in patients with early stage or mild CKD”—a disease which is mainly treated by family doctors/non-nephrologists rather than specialists who have traditionally been considered the center of renal disease treatment. The study aimed to prevent the progression of CKD from an early stage by strictly managing hypertension and diabetes through dietary therapy and by closely coordinating with nephrologists [11]. In this study, we investigated whether there was a synergistic effect with RASi; however, no significant results were found.

### Perspective of Asia

Recent reports in Western populations suggest that RASi may not confer protection against all-cause mortality or preservation of kidney function in patients with non-proteinuric CKD [21, 25]. In this study, we examined whether similar findings are observed in Japanese patients. Further studies are needed to determine whether this trend is consistent across Asian populations.

### Limitations

The first limitation of this study is that the intervention period was limited to 3.5 years, restricting our ability to assess long-term effects. Second, the study population consisted exclusively of Japanese patients, which could limit how broadly our findings can be applied. Third, the sample size was small, which might have limited the robustness of the results. Fourth, advanced confounder control methods were lacking. Finally, due to the lack of data on prior heart failure or cardiovascular events, and 30% of missing values in model 3, we were unable to identify the underlying CKD disease, as the collected data was from primary care physicians, and only a nephrologist can determine the underlying disease.

## Conclusion

In summary, RASi treatment was not linked to a decreased incidence of long-term events or mortality in those with nonproteinuric CKD. Additional research is required to understand the function of RAS blocking in high-risk nonproteinuric CKD patients and to provide individualized therapies for this marginalized demographic.

## Supplementary information

Supplementary information
