# Supplementary material for: Renin-angiotensin system inhibitor use and cardio-renal outcomes in non-proteinuric chronic kidney disease: a post-hoc analysis of the Frontier of Renal Outcome Modification-Japan study
Source: Hypertens Res. 2026 Jan 14;49(4):1161–9. doi: 10.1038/s41440-025-02536-x (PMC13050643; doi:10.1038/s41440-025-02536-x)
Supplement: Supplementary file 1 — Supplementary information [file 41440_2025_2536_MOESM1_ESM.docx]

**SUPPLEMENTARY MATERIAL**

**Supplementary Table S1. Additional clinical and medication data.**

| **Characteristics** | **Overall** | **RASI** | **no RASI** |
| --- | --- | --- | --- |
| Hyperuricemia | 306 (48.9) | 246 (50.4) | 60 (43.5) |
| Creatinine, mg/dL | 1.3 ± 0.5 | 1.3 ± 0.5 | 1.2 ± 0.4 |
| Blood urea nitrogen, mg/dL | 22.4 ± 8.6 | 22.5 ± 8.7 | 21.8 ± 8.2 |
| HDL cholesterol, mg/dL | 53.4 ± 16.5 | 53.9 ± 17.3 | 52.0 ± 13.5 |
| Total cholesterol, mg/dL | 193.5 ± 33.4 | 194.1 ± 33.8 | 191.7 ± 31.9 |
| Triglycerides, mg/dL | 164.3 ± 113.4 | 167.0 ± 121.0 | 154.5 ± 80.0 |
| Uric acid, mg/dL | 6.5 ± 1.7 | 6.6 ± 1.8* | 6.2 ± 1.5 |
| Total protein, g/dL | 7.3 ± 0.5 | 7.3 ± 0.4 | 7.2 ± 0.5 |
| Albumin, g/dL | 4.3 ± 0.3 | 4.3 ± 0.3* | 4.2 ± 0.4 |
| Nitrate, no. (%) | 34 (5.4) | 27 (5.5) | 7 (5.0) |
| Fibrate, no. (%) | 48 (7.6) | 34 (6.9) | 14 (10.0) |
| Ezetimibe, no. (%) | 8 (1.3) | 6 (1.2) | 2 (1.4) |
| Uric acid lowering, no. (%) | 198 (31.4) | 161 (32.9) | 37 (26.4) |
| OHA, no. (%) | 212 (33.7) | 167 (34.1) | 45 (32.1) |
| Insulin, no. (%) | 64 (10.2) | 51 (10.4) | 13 (9.3) |
| Antiplatelet, no. (%) | 176 (27.9) | 139 (28.4) | 37 (26.4) |
| Anticoagulant, no. (%) | 33 (5.2) | 23 (4.7) | 10 (7.1) |
| Vitamin D, no. (%) | 6 (1.0) | 4 (0.8) | 2 (1.4) |

**Supplementary Table S2. The details of cardiovascular events**

| **Outcome** | **Model** | **Hazard Ratio (95% CI)** | **p** |
| --- | --- | --- | --- |
| Stroke |  |  |  |
|  | Model 1 | 1.42 (0.54–3.71) | 0.475 |
|  | Model 2 | 1.63 (0.62–4.28) | 0.322 |
|  | Model 3 | 1.11 (0.36–3.45) | 0.858 |
|  |  |  |  |
| Heart Failure |  |  |  |
|  | Model 1 | 1.85 (0.55–6.24) | 0.323 |
|  | Model 2 | 1.98 (0.58–6.77) | 0.278 |
|  | Model 3 | 2.64 (0.56–12.48) | 0.222 |
|  |  |  |  |
| Myocardial infarction | |  |  |
|  | Model 1 | 0.85 (0.27–2.62) | 0.773 |
|  | Model 2 | 0.80 (0.26–2.53) | 0.71 |
|  | Model 3 | 0.90 (0.17–4.83) | 0.906 |

**Supplementary Table S3. (A) Angiotensin-converting enzyme** **inhibitors** **analysis**

| **Outcome** | **Model** | **ACE inhibitor** | | **no ACE inhibitor** | | **Hazard Ratio (95% CI)** | **p** |
| --- | --- | --- | --- | --- | --- | --- | --- |
|  |  | Total No. | Event No. (%) | Total No. | Event No. (%) |  |  |
| Primary outcome | |  |  |  |  |  |  |
|  | Model 1 | 89 | 19 | 541 | 80 | 1.48 (0.89–2.43) | 0.128 |
|  | Model 2 | 83 | 18 | 526 | 80 | 1.41 (0.84–2.36) | 0.192 |
|  | Model 3 | 54 | 11 | 373 | 53 | 1.38 (0.67–2.85) | 0.377 |
|  |  |  |  |  |  |  |  |
| Secondary oucomes | |  |  |  |  |  |  |
| All cause death | |  |  |  |  |  |  |
|  | Model 1 | 89 | 10 | 541 | 36 | 1.69 (0.84–3.40) | 0.144 |
|  | Model 2 | 83 | 9 | 526 | 36 | 1.48 (0.71–3.09) | 0.302 |
|  | Model 3 | 54 | 7 | 373 | 25 | 1.44 (0.57–3.68) | 0.443 |
|  |  |  |  |  |  |  |  |
| Cardiovascular events | | |  |  |  |  |  |
|  | Model 1 | 89 | 12 | 541 | 54 | 1.37 (0.73–2.56) | 0.327 |
|  | Model 2 | 83 | 11 | 526 | 54 | 1.27 (0.66–2.43) | 0.475 |
|  | Model 3 | 54 | 6 | 373 | 33 | 1.63 (0.65–4.08) | 0.298 |
|  |  |  |  |  |  |  |  |
| Renal replacement therapy | | |  |  |  |  |  |
|  | Model 1 | 89 | 8 | 541 | 28 | 1.74 (0.79–3.81) | 0.168 |
|  | Model 2 | 83 | 8 | 526 | 28 | 1.55 (0.68–3.54) | 0.295 |
|  | Model 3 | 54 | 6 | 373 | 21 | 1.26 (0.37–4.34) | 0.710 |

**(B) Angiotensin receptor blockers** **analysis**

| **Outcome** | **Model** | **ARB** | | **no ARB** | | **Hazard Ratio (95% CI)** | **p** |
| --- | --- | --- | --- | --- | --- | --- | --- |
|  |  | Total No. | Event No. (%) | Total No. | Event No. (%) |  |  |
| Primary outcome | |  |  |  |  |  |  |
|  | Model 1 | 428 | 70 | 202 | 29 | 1.14 (0.74–1.76) | 0.553 |
|  | Model 2 | 419 | 70 | 190 | 28 | 1.20 (0.77–1.87) | 0.413 |
|  | Model 3 | 295 | 45 | 132 | 19 | 1.29 (0.71–2.34) | 0.405 |
|  |  |  |  |  |  |  |  |
| Secondary oucomes | |  |  |  |  |  |  |
| All cause death | |  |  |  |  |  |  |
|  | Model 1 | 428 | 29 | 202 | 17 | 0.81 (0.45–1.48) | 0.499 |
|  | Model 2 | 419 | 29 | 190 | 16 | 0.95 (0.52–1.76) | 0.875 |
|  | Model 3 | 295 | 19 | 132 | 13 | 0.82 (0.38–1.77) | 0.605 |
|  |  |  |  |  |  |  |  |
| Cardiovascular events | | |  |  |  |  |  |
|  | Model 1 | 428 | 48 | 202 | 18 | 1.27 (0.74–2.18) | 0.388 |
|  | Model 2 | 419 | 48 | 190 | 17 | 1.44 (0.83–2.51) | 0.198 |
|  | Model 3 | 295 | 28 | 132 | 11 | 1.29 (0.62–2.69) | 0.498 |
|  |  |  |  |  |  |  |  |
| Renal replacement therapy | | |  |  |  |  |  |
|  | Model 1 | 428 | 25 | 202 | 11 | 1.08 (0.53–2.19) | 0.841 |
|  | Model 2 | 419 | 25 | 190 | 11 | 1.21 (0.59–2.51) | 0.602 |
|  | Model 3 | 295 | 19 | 132 | 8 | 1.95 (0.65–5.87) | 0.237 |

**Supplementary Table S4. (A) All-cause death outcomes in the prespecified subgroups.**

| **Subgroup** | | **RASI** | **no RASI** | **Hazard Ratio (95% CI)** | **p** |
| --- | --- | --- | --- | --- | --- |
|  |  | *no. of patients with event/total no.* | |  |  |
| Age |  |  |  |  |  |
|  | <65 | 8/232 | 1/49 | 1.47 (0.18–11.7) | 0.759 |
|  | >=65 | 32/295 | 8/99 | 1.57 (0.72–3.41) | 0.349 |
| Sex |  |  |  |  |  |
|  | Male | 13/159 | 1/44 | 3.66 (0.48–27.9) | 0.211 |
|  | Female | 27/368 | 8/104 | 0.96 (0.44–2.12) | 0.696 |
| DM |  |  |  |  |  |
|  | No | 21/264 | 6/74 | 1.03 (0.42–2.56) | 0.940 |
|  | Yes | 19/261 | 3/72 | 1.76 (0.52–5.95) | 0.455 |
| BMI |  |  |  |  |  |
|  | <25 | 25/280 | 2/62 | 2.73 (0.65–11.5) | 0.196 |
|  | >=25 | 14/235 | 7/79 | 0.69 (0.28–1.71) | 0.317 |
| eGFR | |  |  |  |  |
|  | <45 | 19/234 | 4/56 | 1.32 (0.45–3.88) | 0.722 |
|  | >=45 | 21/293 | 5/92 | 1.20 (0.45–3.18) | 0.842 |
| Albumin | |  |  |  |  |
|  | < 4.3 | 17/172 | 4/62 | 1.63 (0.55–4.85) | 0.424 |
|  | >= 4.3 | 13/224 | 3/55 | 0.99 (0.28–3.48) | 0.761 |
| K |  |  |  |  |  |
|  | <5.5 | 35/463 | 8/130 | 1.25 (0.58–2.69) | 0.747 |
|  | >=5.5 | 4/25 | 0/6 | NA | NA |
| Systolic BP | |  |  |  |  |
|  | <130 | 14/194 | 2/68 | 2.53 (0.57–11.1) | 0.261 |
|  | >=130 | 26/331 | 7/78 | 0.90 (0.39–2.07) | 0.669 |
| Diastolic BP | |  |  |  |  |
|  | <80 | 27/312 | 5/103 | 1.88 (0.72–4.89) | 0.217 |
|  | >=80 | 13/213 | 4/43 | 0.63 (0.21–1.94) | 0.272 |

**(B)** **Cardiovascular event outcomes in the prespecified subgroups**.

| **Subgroup** | | **RASI** | **no RASI** | **Hazard Ratio (95% CI)** | **p** |
| --- | --- | --- | --- | --- | --- |
|  |  | *no. of patients with event/total no.* | |  |  |
| Age |  |  |  |  |  |
|  | <65 | 13/232 | 3/49 | 0.81 (0.23–2.85) | 0.680 |
|  | >=65 | 44/295 | 8/99 | 2.09 (0.98–4.44) | 0.065 |
| Sex |  |  |  |  |  |
|  | Male | 21/159 | 0/44 | NA | NA |
|  | Female | 36/368 | 11/104 | 0.92 (0.47–1.81) | 0.711 |
| DM |  |  |  |  |  |
|  | No | 16/264 | 4/74 | 1.17 (0.39–3.51) | 0.901 |
|  | Yes | 41/261 | 7/72 | 1.66 (0.75–3.70) | 0.257 |
| BMI |  |  |  |  |  |
|  | <25 | 29/280 | 4/62 | 1.56 (0.55–4.45) | 0.403 |
|  | >=25 | 27/235 | 7/79 | 1.35 (0.59–3.09) | 0.580 |
| eGFR | |  |  |  |  |
|  | <45 | 20/234 | 3/56 | 1.82 (0.54–6.14) | 0.357 |
|  | >=45 | 37/293 | 8/92 | 1.39 (0.65–2.98) | 0.467 |
| Albumin | |  |  |  |  |
|  | < 4.3 | 19/172 | 5/62 | 1.49 (0.56–4.00) | 0.406 |
|  | >= 4.3 | 19/224 | 3/55 | 1.53 (0.45–5.18) | 0.623 |
| K |  |  |  |  |  |
|  | <5.5 | 47/463 | 11/130 | 1.22 (0.63–2.35) | 0.648 |
|  | >=5.5 | 3/25 | 0/6 | NA | NA |
| Systolic BP | |  |  |  |  |
|  | <130 | 23/194 | 4/68 | 1.97 (0.68–5.69) | 0.277 |
|  | >=130 | 34/331 | 7/78 | 1.19 (0.53–2.69) | 0.644 |
| Diastolic BP | |  |  |  |  |
|  | <80 | 36/312 | 9/103 | 1.34 (0.64–2.79) | 0.513 |
|  | >=80 | 21/213 | 2/43 | 2.19 (0.51–9.36) | 0.303 |

**(C) Renal replacement therapy outcomes in the prespecified subgroups.**

| **Subgroup** | | **RASI** | **no RASI** | **Hazard Ratio (95% CI)** | **p** |
| --- | --- | --- | --- | --- | --- |
|  |  | *no. of patients with event/total no.* | |  |  |
| Age |  |  |  |  |  |
|  | <65 | 13/232 | 1/49 | 2.31 (0.30–17.7) | 0.631 |
|  | >=65 | 24/295 | 5/99 | 1.85 (0.71–4.85) | 0.372 |
| Sex |  |  |  |  |  |
|  | Male | 11/159 | 3/44 | 1.04 (0.29–3.72) | 0.796 |
|  | Female | 26/368 | 3/104 | 2.45 (0.74–8.09) | 0.274 |
| DM |  |  |  |  |  |
|  | No | 22/264 | 4/74 | 1.59 (0.55–4.61) | 0.588 |
|  | Yes | 15/261 | 2/72 | 2.10 (0.48–9.17) | 0.530 |
| BMI |  |  |  |  |  |
|  | <25 | 25/280 | 3/62 | 1.77 (0.54–5.87) | 0.542 |
|  | >=25 | 12/235 | 3/79 | 1.38 (0.39–4.89) | 0.873 |
| eGFR | |  |  |  |  |
|  | <45 | 34/234 | 6/56 | 1.56 (0.65–3.71) | 0.599 |
|  | >=45 | 3/293 | 0/92 | NA | NA |
| Albumin | |  |  |  |  |
|  | < 4.3 | 19/172 | 3/62 | 2.42 (0.72–8.19) | 0.360 |
|  | >= 4.3 | 11/224 | 1/55 | 2.58 (0.33–20.0) | 0.492 |
| K |  |  |  |  |  |
|  | <5.5 | 34/463 | 6/130 | 1.61 (0.67–3.82) | 0.603 |
|  | >=5.5 | 2/25 | 0/6 | NA | NA |
| Systolic BP | |  |  |  |  |
|  | <130 | 15/194 | 4/68 | 1.30 (0.43–3.91) | 0.982 |
|  | >=130 | 22/331 | 2/78 | 2.68 (0.63–11.4) | 0.293 |
| Diatolic BP | |  |  |  |  |
|  | <80 | 25/312 | 5/103 | 1.71 (0.65–4.47) | 0.465 |
|  | >=80 | 12/213 | 1/43 | 2.37 (0.31–18.3) | 0.603 |

**Supplementary Table S5. (A) Effect of suppressing the estimated glomerular filtration rate slope**.

|  | *Model 1 | | |  | **Model 2 | | |  | ***Model 3 | | |
| --- | --- | --- | --- | --- | --- | --- | --- | --- | --- | --- | --- |
|  | Odds ratio | 95% CI | *p* |  | Odds ratio | 95% CI | *p* |  | Odds ratio | 95% CI | *p* |
| RASI | 1.707 | 0.854–3.546 | 0.138 |  | 1.647 | 0.788–3.574 | 0.193 |  | 1.632 | 0.678–4.167 | 0.286 |
| Age |  |  |  |  | 0.979 | 0.938–1.021 | 0.328 |  | 0.989 | 0.941–1.039 | 0.650 |
| Sex |  |  |  |  | 0.720 | 0.377–1.355 | 0.313 |  | 0.591 | 0.242–1.394 | 0.237 |
| DM |  |  |  |  | 1.997 | 1.089–3.725 | 0.027 |  | 1.928 | 0.919–4.152 | 0.087 |
| BMI |  |  |  |  | 1.005 | 0.927–1.09 | 0.904 |  | 0.972 | 0.880–1.073 | 0.573 |
| eGFR slope | |  |  |  | 0.962 | 0.934–0.989 | 0.008 |  | 0.940 | 0.903–0.977 | 0.002 |
| Alb |  |  |  |  |  |  |  |  | 0.907 | 0.341–2.678 | 0.847 |
| Hb |  |  |  |  |  |  |  |  | 0.968 | 0.766–1.224 | 0.787 |
| K |  |  |  |  |  |  |  |  | 0.683 | 0.360–1.263 | 0.230 |
| Statin |  |  |  |  |  |  |  |  | 1.487 | 0.737–3.028 | 0.269 |
| MRA |  |  |  |  |  |  |  |  | NA | NA | NA |

**(B) The effect of suppressing the new onset of proteinuria.**

|  | *Model 1 | | |  | **Model 2 | |  | | | ***Model 3 | | |
| --- | --- | --- | --- | --- | --- | --- | --- | --- | --- | --- | --- | --- |
|  | β | 95% CI | *p* |  | β | 95% CI | | *p* |  | β | 95% CI | *p* |
| RASI | 0.015 | -0.573–0.604 | 0.959 |  | -0.115 | -0.727–0.498 | | 0.714 |  | -0.180 | -0.18 (-0.849–0.488) | 0.596 |
| Age |  |  |  |  | -0.025 | -0.059–0.010 | | 0.158 |  | -0.005 | -0.005 (-0.044–0.034) | 0.802 |
| Sex |  |  |  |  | -0.285 | -0.832–0.263 | | 0.308 |  | 0.319 | 0.319 (-0.363–1.001) | 0.358 |
| DM |  |  |  |  | 0.133 | -0.37–0.635 | | 0.604 |  | 0.409 | 0.409 (-0.162–0.979) | 0.160 |
| BMI |  |  |  |  | -0.031 | -0.097–0.036 | | 0.362 |  | -0.059 | -0.059 (-0.135–0.018) | 0.132 |
| eGFR slope | |  |  |  | -0.004 | -0.027–0.019 | | 0.754 |  | -0.031 | -0.031 (-0.061–0.002) | 0.035 |
| Alb |  |  |  |  |  |  | |  |  | 1.316 | 1.316 (0.45–2.182) | 0.003 |
| Hb |  |  |  |  |  |  | |  |  | 0.281 | 0.281 (0.096–0.467) | 0.003 |
| K |  |  |  |  |  |  | |  |  | -0.187 | -0.187 (-0.704–0.331) | 0.479 |
| Statin |  |  |  |  |  |  | |  |  | 0.374 | 0.374 (-0.219–0.967) | 0.216 |
| MRA |  |  |  |  |  |  | |  |  | -0.122 | -0.122 (-1.808–1.564) | 0.887 |

**Supplementary Table S6. Multivariate analysis revealed high blood pressure and a higher proportion of people using calcium channel blockers.**

| **Outcome** | **Hazard Ratio (95% CI)** | **P** |
| --- | --- | --- |
| Primary outcome | 1.20 (0.60–2.40) | 0.601 |
| Secondary oucomes |  |  |
| All cause death | 1.02 (0.41–2.53) | 0.966 |
| Cardiovascular events | 0.87 (0.37–2.07) | 0.758 |
| Renal replacement therapy | 1.86 (0.56–6.20) | 0.311 |

**Supplementary Table S7. Multivariate analysis revealed a synergistic effect of early intervention with renin-angiotensin system inhibitors.**

| **Outcome** | **Model** | **Hazard Ratio (95% CI)** | **p** |
| --- | --- | --- | --- |
| Primary outcome | |  |  |
|  | Model 1 | 2.12 (0.75–6.00) | 0.156 |
|  | Model 2 | 2.07 (0.73–5.91) | 0.173 |
|  | Model 3 | 1.52 (0.50–4.63) | 0.465 |
|  |  |  |  |
| Secondary oucomes | |  |  |
| All cause death | |  |  |
|  | Model 1 | 5.34 (0.72–39.82) | 0.102 |
|  | Model 2 | 6.27 (0.83–47.43) | 0.075 |
|  | Model 3 | NA | NA |
|  |  |  |  |
| Cardiovascular events | |  |  |
|  | Model 1 | 2.03 (0.46–8.89) | 0.346 |
|  | Model 2 | 1.91 (0.42–8.62) | 0.402 |
|  | Model 3 | 1.13 (0.22–5.81) | 0.885 |
|  |  |  |  |
| Renal replacement therapy | |  |  |
|  | Model 1 | 2.35 (0.55–10.14) | 0.251 |
|  | Model 2 | 2.59 (0.59–11.26) | 0.205 |
|  | Model 3 | 2.29 (0.46–11.46) | 0.313 |
